# A survey of the knowledge and practices of nursing students of Mbarara University of Science and Technology around Monitoring Fluid Requirements for burns patients on surgical ward at Mbarara Regional Referral Hospital

**DOI:** 10.1186/s12912-022-01041-7

**Published:** 2022-09-21

**Authors:** Joan Atuhaire, Jonathan Kajjimu, Javilla Kakooza Kamya, Grifance Opio, Frank Lubega, Reagan Kakande, William Mwanje, Andrew Tagg

**Affiliations:** 1 Faculty of Medicine, Mbarara University of Science and Technology, P. O. Box 1410, Mbarara, Uganda; 2 Emergency Department, Western Health, Melbourne, Australia

**Keywords:** Fluid balance, Fluid monitoring, Nursing students, Burns, Uganda

## Abstract

**Background:**

There is a high mortality of burns especially in low- and middle-income countries which already have less developed healthcare systems. Besides, little is known about nursing students’ knowledge and practices towards the need to monitor fluid requirements in admitted burns patients.

**Objective:**

To assess the knowledge and practices of nursing students regarding monitoring fluid requirements for hospitalised paediatrics and adult burn patients on the surgical ward at Mbarara regional referral hospital.

**Methods:**

We conducted an online descriptive cross-sectional study among clinical nursing students at Mbarara University of Science and Technology (MUST) during September and November 2021. We assessed knowledge and practices using an adapted questionnaire. Summary statistics were then used to describe the data.

**Results:**

Thirty-seven nursing students (64.9% response rate) participated in our survey. Twenty one (56.8%) were female and had a modal age range of 20-24 years. Nineteen (51.4%) of the students were BNC (Bachelor of Nursing Completion) students, with the rest being BNS (Bachelor of Nursing). More than 75% of students correctly answered each of two out of the ten questions. More than three quarters of the students reported having done each of six out of the eleven practices surveyed.

**Conclusion:**

Nursing students had poor knowledge and fair satisfactory practices regarding monitoring of the fluid requirements in burns patients. More similar studies are needed to survey more nursing students on this topic and to henceforth evaluate the need for periodic re-trainings and reassessment of clinical skills of nursing students.

**Supplementary Information:**

The online version contains supplementary material available at 10.1186/s12912-022-01041-7.

## Introduction

Burns present a serious public health threat causing an estimated 180,000 deaths annually with majority of these occurring in low- and middle-income countries [1]. In 2018, nearly 11 million people were burned severely enough to require medical attention [2]. According to a scoping review by Mukagaju and colleagues, between 1993 and 2019, 44,369 patients with burns were reported in East Africa [3]. Mortality of burns has declined in the past decades but people living in the middle- and low-income countries still face a challenge [4]. Despite the high incidence of burns in sub-Saharan Africa, health care providers skilled in burns care are rare, and there is a need to scale up proper training to manage such patients [5]. Management of burns patients involves both immediate care and long-term care. The initial management requires prioritization of the airway, breathing, and circulation, with the goals of long-term care including closure of the wound, management of the hypermetabolic response, and prevention of infection and multiple organ dysfunction [1]. It is widely accepted that fluid loss is one of the major problems faced following major burns. Appropriate fluid management directly improves the prognosis and outcome of burns patients [6]. According to the American Burn Association guidelines, adult and paediatrics patients with burns of greater than 20% total body surface area (TBSA), should receive fluid resuscitation based on their weight and surface area burnt [7, 8].

However, little is known about the knowledge and understanding of nursing students towards appropriate fluid resuscitation monitoring. A recent experimental study found a health education program to improve nurses’ knowledge surrounding fluid and electrolyte replacement [9].

Inaccurate monitoring of fluid balance, especially in burns patients, may worsen the patients’ condition. This study aimed to examine the knowledge and practices of clinical nursing students at MUST around the monitoring of adult burns’ patients in the surgical ward of Mbarara regional referral hospital (MRRH).

## Methods

### Study design

We conducted an online based cross-sectional study with a quantitative research methods approach.

### Study area

The study was conducted at Mbarara University of Science and Technology, among nursing students who had rotated on the surgical ward of Mbarara Regional Referral Hospital (MRRH). Mbarara University, is a public university in Uganda. Student intake and instruction commenced in 1989. It is one of the eight public universities and degree-awarding institutions in the country and is accredited by the Uganda National Council of Science and Technology.

### Target population

Bachelor of Nursing students and Bachelor of Nursing Completion students.

### Study population

The study participants included Bachelor of nursing (BNS) and Bachelor of Nursing Completion (BNC) students studying at MUST. BNC and BNS students’ study for 2 and 4 years respectively at MUST and start their clinical years in year one and two respectively. Our targeted population comprised approximately 60 students. They were chosen because they have studied the management of burns, and they have managed burns patients during their clinical studies.

### Sample size determination

We determined the sample size using Yamane Taro’s method (Yamane, 1967:886) of sample size determination which assumes a 95% level of confidence and a 0.5 *p* value.

Yamane’s formula,

$$\mathrm{n}=\mathrm{N}/1+\mathrm{N}\ {\left(\mathrm{e}\right)}^2$$

Where;

- *n* = sample size
- *N* = total population which is 60.
- e = margin of error = 0.05; the level of precision or level of significance.
- *n* = 60/ 1 + 60 (0.05)^2^
- *n* = 52 study participants
- 10% compensation for those the researcher may not get (non-correspondence) is 5 participants; from 52+ (10% of 52),

Therefore, the total sample size was 57 participants.

### Sampling strategy

A consecutive sampling technique was used to enrol participants in the study.

### Inclusion criteria

- Bachelor of Nursing students in the clinical years (years 3 and 4) and the Bachelor of Nursing completion students who have studied burns.
- And students who consented to take part in the study.

### Exclusion criteria

- Students offline during the data collection period.

### Data collection tools

The research instrument was composed of a self-administered questionnaire with questions that were adapted from a recent study by Awad and colleagues [9]. A panel of experts from our Department of Nursing including our research supervisor, did content validity of the questionnaire and found all questionnaire items to be adequately measuring the construct intended to assess and to sufficiently measure the domain of interest. The questionnaire was hosted using Google forms. It consisted of both open and close-ended questions. The questionnaire had three sections A, B, and C (See Additional file 1). Section A collected students’ socio-demographics, while section B (10 questions) and section C (11 questions) consisted of an assessment of the students’ knowledge and practices in regards to monitoring of fluid requirements among burn patients respectively.

### Scoring system

A scoring system adapted along with the questionnaire was used to provide scores calculated basing on students’ responses. Each question received a binary answer (0 or 1). Scores of greater than 75% were considered good; Scores between 50 and 75% were considered average; and scores less than 50% were considered poor.

We also assessed practical exposure. Each practice question received a binary answer (0 or 1), basing on whether students had or had not done a given practice, i.e. (1) = Done, while (0) = Not done. The total practices were considered satisfactory if the total score of a given practice was ≥75%, and considered unsatisfactory if it is less than 75%.

### Data collection procedure

Researchers shared a Google form link with the questionnaire to potential participants in their class WhatsApp groups. Digital informed consent was obtained from all participants prior to participation. Data was collected from November 8, 2021 till December 6, 2021 (4 weeks).

### Quality control

We conducted a pilot study on 10% of the sample size outside the study population (five nursing students from Bishop Stuart University) to ensure that the tool was reliable and valid. For validity of our tool, we had a panel of experts that measured the content validity of our questionnaire and found all questionnaire items to be adequately measuring the construct intended to assess. To test reliability of our tool, we calculated a Cronbach alpha of 0.71 using our pilot study data.

### Data management, and analysis

Data was collected using Google forms, and extracted as an Excel file. Before analysis, it was checked. No duplicates or incomplete entries were found. We used STATA version 14 for analysis. We used descriptive statistics to inform frequency and percentage levels. Analysed data was then presented in the form of tables, and pie charts.

## Results

Overall, we received 37 responses (64.9% response rate). Most students were aged 20 -24 years and more than half of the students were female (21, 56.7%) and pursuing BNC (19, 51.4%). 64.9% of the students surveyed were single. The majority of the students (23, 53.2%) had spent more than one semester on a surgical ward (See Table 1).

**Table 1:** Characteristics of study participants

***Socio-demographics***	***Freq.***	***Percent (%)***	***Mean***	***SD***
***Age***				
*20-24*	17	45.95		
*25-29*	4	10.81		
*30-34*	9	24.32		
*35-39*	4	10.81		
*40-44*	2	5.41		
*44+*	1	2.70		
***Gender***				
*Female*	21	56.76		
*Male*	16	43.24		
***Program***				
*BNC*	19	51.35		
*BNS*	18	48.65		
***Year of study***			2.73	± 0.99
*1*	3	8.11		
*2*	15	40.54		
*3*	8	21.62		
*4*	11	29.73		
***Semesters rotated on surgical ward***			1.95	± 0.85
*1*	14	37.84		
*2*	11	29.73		
*3*	12	32.43		
***Previous training on fluids and electrolytes follow-up for burns patients***				
*Yes*	20	45.95		
*No*	17	54.05		

### Knowledge of nursing students regarding fluid monitoring for burns patients

Students had insufficient knowledge of fluid monitoring for burns patients. Students demonstrated good knowledge (> 75% students correctly answering a given question) for only two out of ten survey questions. 16.2% of the students were able to correctly state Parklands’ formula and 29.7% of the students knew the importance of fluid replacement during the initial phase of resuscitation (More details in Table 2).

**Table 2:** Distribution of students regarding their knowledge about fluids for burns patients

Students’ Knowledge	Correct answer		Incorrect answer	
	Freq.	Percent (%)	Freq.	Percent (%)
The normal range (%) of body fluids in adults	27	72.97	10	27.03
The meaning of fluid balance in body	21	56.76	16	43.24
If body systems should be monitored to assess the fluid balance	33	89.19	4	10.81
The nurse’s role in assessing the urinary system of a burns patient	37	100	–	–
The nurse’s role in assessing the cardiovascular system of a burns patient	9	24.32	28	75.68
The meaning of fluids replacement	24	64.86	13	35.14
The basic steps in fluid replacement process	5	13.51	32	86.49
Importance of fluid replacement in burns patients during the initial phase	11	29.73	26	70.27
Stating Parkland’s formula used in fluid replacement.	6	16.22	31	83.78
Amount of fluid using the Parkland formula to be given to a patient weighing 30 kg in the resuscitative phase, with 28% TBSA	18	48.65	19	51.35

### Practices of nursing students regarding monitoring fluid requirements for burns patients

Overall, nursing students had fair practices. Students demonstrated satisfactory practices (≥ 75% students reporting have done a given practice) in six out of eleven practices surveyed. All participants reported performing hand hygiene during caring for burns patients in their fluid replacement stages. A significant big proportion of participants documented any fluids and additives given to patients while on ward (See Table 3).

**Table 3:** Distribution of students regarding their Practices towards monitoring of fluids in burned patients within the first 24–72 h of admission

Students’ practices	Done		Not done	
	Freq.	Percent (%)	Freq.	Percent (%)
Performing hand hygiene	37	100	–	–
Checking to see if IV line flows freely	35	94.59	2	5.41
Assessing patients using rule of nines.	26	70.27	11	29.73
Calculating fluids using parklands formula	23	62.16	14	37.84
Check the infusion rate \date.	32	86.49	5	13.51
Document the prescribed fluid on the patients chart.	34	91.89	3	8.11
Check if inflammation signs show.	26	70.27	11	29.73
Record the time when the administration of fluid starts	31	83.78	6	16.22
Mentioning the amount of fluid infused and documenting the additives that were added to the fluid	26	70.27	11	29.73
Record date and your signature after administering fluids.	34	91.89	3	8.11

## Discussion

This study was designed to assess the knowledge and practices of MUST nursing students around fluid monitoring in hospitalised burns patients on the surgical ward at MRRH. We generally found that nursing students had insufficient knowledge and poor practices. This is the first study looking at the knowledge and practices of nursing students regarding monitoring of fluid requirements for adult burns patients in Uganda. The findings of our study are consistent with pre-intervention findings of prior similar studies [9, 10]. These found poor knowledge regarding monitoring of fluid replacement for burns patients among nurses prior to enrollment in interventional educational program.

Three quarters of the students managed to pass each of the two out of ten knowledge questions concerning monitoring fluid replacement of burns patient. Only 13.51% of the participants knew the basic steps in the fluid replacement process. Given that knowledge of fluid balance is very important in guiding nurses when caring for critically ill patients [11], our identified knowledge gap, warrants further study as students with insufficient knowledge may cause greater harm than benefit to patients.

When looking at fluid administration our study produced results that corroborate previous studies. These found nurses have difficulty in using formulae for fluid replacement [9, 12]. Parkland’s formula is a commonly used method to estimate the amount of fluid required to adequately resuscitate burns patients in the first 24 hours of acute burn injury basing on their burnt estimated total body surface [13]. 16.2% of our study participants were able to correctly state Parklands’ formula, while 48.7% of the students were able to select the right fluid requirement in a set scenario using the Parkland formula. Some students may have correctly guessed an answer to the scenario presented to them, producing contradicting results. Interventional studies might address this knowledge gap.

In our study, we could not completely confirm this but we think lack of prior training around fluid and electrolyte replacement for burns patients and the limited number of semesters rotated on surgical ward, one semester (14, 37.8%) and 2 semesters (11, 29.7%), may be the cause of lack of sufficient knowledge amongst surveyed students. Further research will be needed to confirm or disapprove this.

In contrast, Kanakalakshmi [14], found more than one third of the working nurses followed safe practices regarding fluid and electrolyte replacement therapy. Our study found a significant proportion of students displaying safe practices regarding fluid replacement for burns patients. More than three quarters of students reported having performed each of six out of the eleven safe practices. There is a need to observe these students and assess to see if these practices were performed correctly.

However, with a small sample size, caution must be taken. Our findings may not be transferable to nursing students at Mbarara University and in Uganda generally.

We were unable to clearly determine the degree of burns which our participants had taken care of.

The aim of this study was to determine the knowledge and practices of nursing students around fluid monitoring in burns patients. Despite its descriptive nature, our investigation has shown that nursing students have poor knowledge and fair practices regarding fluid replacement in this patient cohort. Teaching institutions should have periodic surveys of their students’ clinical skills to ensure that they are following appropriate guidelines. In-order to improve the survival rates of burn patients, appropriate fluid management of major burns is crucial.

### Strengths and limitations of the study

To the best of our knowledge, this is the first study to address the knowledge and practices for nursing students towards monitoring of fluid replacement in burn patients in Uganda.

With a small sample size and low response rate to our survey, caution must be taken as the findings may not be generalizable to nursing students at Mbarara University or in Uganda generally. This limited us from achieving a true picture of the knowledge and practices of nursing students at Mbarara university towards monitoring of fluid requirements for burns patients on surgical ward at Mbarara Regional Referral Hospital.

Additionally, we were unable to determine whether students performed the required practices correctly as we subjectively accessed students’ skills due to limited resources.

Some students were offline during time of data collection. However, the researchers believe that data from this study will lay a good foundation for future scholars interested in studying the topic.

## Conclusion

The aim of this study was to assess the level of knowledge and the practices of nursing students around fluid replacement in burns patients. Despite its descriptive nature, our investigation has shown that nursing students surveyed at MUST have poor knowledge and fair practices regarding fluid replacement in this patient cohort.

## Recommendations

There is need for supplementary fluid balance monitoring and replacement educational trainings designed to help hone the clinical skills of nursing students that responded to our survey.

We recommend that more teaching institutions in Uganda to survey clinical skills of their nursing students in order to confirm or refute findings of our survey.

## Supplementary Information

**Additional file 1.** Questionnaire.

**Additional file 2.** Data set.

## Data Availability

All data generated or analysed during this study are included in this published article [and its supplementary information files].
