# Supplementary material for: A survey of the knowledge and practices of nursing students of Mbarara University of Science and Technology around Monitoring Fluid Requirements for burns patients on surgical ward at Mbarara Regional Referral Hospital
Source: BMC Nurs. 2022 Sep 21;21:258. doi: 10.1186/s12912-022-01041-7 (PMC9490969; doi:10.1186/s12912-022-01041-7)
Supplement: Supplementary file 1 — Additional file 1. Questionnaire. [file 12912_2022_1041_MOESM1_ESM.pdf]

Questionnaire

SECTION A: Socio-demographics

Age (Years)

20 - 24

25 - 29

30 - 34

35 - 39

40 - 44

44+

Gender

Male

Female

Religion

Roman Catholic

Anglican

Muslim

Orthodox

Pentecostal

Traditional

None

Program

BNC

BNS

Year of study

1

2

3

4

Tuition funding:

Government-funded

NGO funded

Private funded

Self-funded

Marital status:

Single

Married

Semesters rotated on surgical ward:

1

2

3

Previous training about fluids and electrolytes follow up for burns patients:

Yes

No

SECTION B: Knowledge of nursing students regarding monitoring of fluid requirements for severe burn patients

What is the normal range of body fluids in adults?

A. 60-70
B. 40-60
C. 20-30
D. 45-75

What is the meaning of fluids balance in body?

A. Balance of input and output of fluids inthe body to allow metabolic process tofunction correctly.
B. Amount of fluids taken in and out of thebody
C. Amount of fluids in the intracellular andextracellular compartments.

Body systems should be monitored to assess the fluid balance?

A. Yes

B. No

What is the nurses' role in assessing the urinary system of a burns' patients?

A. Assess and monitor the patient's urineoutput.
B. To rule out any possible cormobidurinary diseases.

What is nurses' role in assessing the cardiovascular system of a burns' patient?

A. Take detailed history of thecardiovascular system.
B. Understand various functions of thecardiovascular system
C. Take an overview of the structure of theheart
D. Monitor the cardiovascular system.

What is the meaning of fluids replacement?

A. Replacement of fluids that are lost fromthe body because of surgery, injury, trauma,dehydration, disease or otherconditions.
B. ts a medical practice of replenishingbodily fluid lost through sweating,bleeding, fluid shifts or otherpathological processes.

What are the basic steps in fluid replacement process?

A. Routine maintainance
B. Optimisation
C. Resuscitation
D. Reassessment
E. Replacement and redistribution

What is the importance of fluid replacement in burns patients during the initial phase?

A. Prevent excessive elevations in heartrate and body temperature
B. Maintain tissue perfusion during burnshock.
C. Maintain circulatory volume.
D. Prevent burn shock

State (Parkland formula) used in fluid replacement.

How much fluid using the Parkland formula should be given to a patient weighing 30kg in the resuscitative phase, with 28% TBSA?

A. 1680mls
B. 2650mls
C. 3360mls
D. 1200mls

SECTION C: Practices of nursing students towards monitoring of fluids in severe burned patients

(Done/Not done responses)

1. Perform hand hygiene.
2. Check I.V line flows freely
3. Assess patients using rule of nine.
4. Calculate fluid using parkland formula.
5. Check the infusion rate \date.
6. Document the prescribed fluid on the chart.
7. Check if inflammation signs show
8. Record the time when the administration of fluid starts
9. Mentioning the amount of fluid infused and documents the additives that were added to the fluid.
10. Record date and signature after administering fluids.
